# Highly Pathogenic Influenza A(H5N1) Virus Survival in Complex Artificial Aquatic Biotopes

**DOI:** 10.1371/journal.pone.0034160

**Published:** 2012-04-13

**Authors:** Viseth Srey Horm, Ramona A. Gutiérrez, John M. Nicholls, Philippe Buchy

**Affiliations:** 1 Virology Unit, Institut Pasteur du Cambodge, Réseau International des Instituts Pasteur, Phnom Penh, Cambodia; 2 Department of Pathology, University of Hong Kong, Pokfulam, Hong Kong, Hong Kong SAR; University of Georgia, United States of America

## Abstract

**Background:**

Very little is known regarding the persistence of Highly Pathogenic Avian Influenza (HPAI) H5N1 viruses in aquatic environments in tropical countries, although environmental materials have been suggested to play a role as reservoirs and sources of transmission for H5N1 viruses.

**Methodology/Principal Findings:**

The survival of HPAI H5N1 viruses in experimental aquatic biotopes (water, mud, aquatic flora and fauna) relevant to field conditions in Cambodia was investigated. Artificial aquatic biotopes, including simple ones containing only mud and water, and complex biotopes involving the presence of aquatic flora and fauna, were set up. They were experimentally contaminated with H5N1 virus. The persistence of HPAI H5N1 virus (local avian and human isolates) was determined by virus isolation in embryonated chicken eggs and by real-time reverse-polymerase chain reaction. Persistence of infectious virus did not exceed 4 days, and was only identified in rain water. No infectious virus particles were detected in pond and lake water or mud even when high inoculum doses were used. However, viral RNA persisted up to 20 days in rain water and 7 days in pond or lake water. Viral RNA was also detected in mud samples, up to 14 days post-contamination in several cases. Infectious virus and viral RNA was detected in few cases in the aquatic fauna and flora, especially in bivalves and labyrinth fish, although these organisms seemed to be mostly passive carriers of the virus rather than host allowing virus replication.

**Conclusions/Significance:**

Although several factors for the survival and persistence of HPAI viruses in the environment are still to be elucidated, and are particularly hard to control in laboratory conditions, our results, along with previous data, support the idea that environmental surveillance is of major relevance for avian influenza control programs.

## Introduction

The highly pathogenic avian influenza (HPAI) H5N1 virus is a major public health concern in Southeast Asia, where it has widely spread since its first detection in 1997 [1] and become enzootic in the region. Cambodia is one of the enzootic countries in tropical areas which has reported a high fatality rate in humans (approximately 90%) [2]. Since the first detection of HPAI H5N1 virus in poultry in 2004 and the first human cases of H5N1 virus infection in 2005, 32 H5N1 outbreaks in poultry and 18 human cases (16 fatalities) of H5N1 infection have occurred up to now [2], [3]. Direct contact with infected poultry is the main source for human contamination. However, previous studies provide additional evidence suggesting bathing or swimming in ponds as a risk factor for human H5N1 contamination [4]. The H5N1 virus has been shown to have the ability to persist outside the host, especially in water [5]-[9] and H5N1 viral RNA was previously detected in environmental specimens, including in the surroundings of H5N1 outbreaks areas in Cambodia [10]. Previous studies have described the survival of H5N1 virus in water, soil or various surfaces in laboratory-controlled conditions with temperatures usually ranging from 0 to 25°C [5], [8], [11], [12] but very little is known regarding the persistence of the virus in environment materials such as surface water, mud, soil in tropical countries where average temperatures can reach over 35°C in the shade. Data on the ability of HPAI H5N1 viruses to remain infective outside of the host is very limited. There also are very few reports discussing the role of aquatic fauna in the transmission cycle of the H5N1 virus. An experimental study conducted with low pathogenic avian influenza (LPAI) demonstrated that Asian clams (*Corbicula fluminea*) were capable of removing and reducing the infectivity of avian influenza viruses (AIVs) in water [13]. On the other hand, a study by Stumpf et al. showed that zebra mussels (*Dreissena polymorpha*) were able to accumulate LPAI virus from the surrounding water and to retain the virus in their bodies over an extended period of time before releasing the virus back into freshwater [14]. These few studies seem to emphasize the need for more relevant data on the survival of HPAI H5N1 virus in natural aquatic environments, including in the presence of aquatic fauna.

Our objectives in this study were: (1) to describe the survival of H5N1 virus in water and mud in experimental setting reproducing as faithfully as possible natural conditions observed in tropical countries; (2) to determine whether aquatic animals such as fish, tadpoles, clams, snails, mussels and aquatic flora may be contaminated and play a role in the persistence of H5N1 virus in water; (3) to determine whether autochthonous aquatic organisms such as bivalves (fresh water mussels) and labyrinth fish (fighting fish) could transmit the virus to each other.

## Materials and Methods

### BioSafety statement

All experiments using HPAI H5N1 virus and all animal experiments were performed within the Biosafety Level 3 (BSL-3) laboratory of the Institut Pasteur in Cambodia (IPC), complying with the Animal Committee regulations of Institut Pasteur in Paris, France, in accordance with the EC 86/609/CEE directive, and approved by the Animal Ethics Committee of Institut Pasteur in Cambodia (permit number: AEC/IPC/002/2008).

### Virus

The clade 1, genotype Z, HPAI H5N1 viruses A/Cambodia/408008/2005 (GenBank accession numbers: HQ664938 to HQ664945) and A/chicken/Cambodia/LC1AL/2007 (GenBank accession numbers: HQ200574 to HQ200581) were used to conduct these experiments. The virus stock was obtained after propagation in Specific Pathogen Free (SPF) 9-to-11-day-old embryonated hen eggs, kindly provided by the National Veterinary Research Institute of Cambodia (NaVRI), Ministry of Agriculture, Forestries and Fisheries (MAFF). The amnio-allantoic fluid (AAF) from the second passage on SPF eggs was harvested 48 hours after inoculation and stored at −80°C until further use. Virus titre was determined by calculating the 50% egg infectious dose (EID50) per mL of virus stock. Titration endpoints were calculated using the method of Reed and Muench [15].

### Mud and water

Mud and water used in the artificial aquatic settings described below were collected from 2 different ponds (with the landlord's official authorization) and from a lake in areas of Kampong Cham province where an H5N1 virus outbreak had previously occurred. Some of the experiments also involved the use of rain water which was collected and stored in big jars within the IPC external facilities until use. Temperature, pH and conductivity results of all water samples were recorded on site at the time of collection. Additional physico-chemical analyses and all microbiological tests were carried out upon arrival at the laboratory (Table S1). All water and mud samples were transported at ambient temperature (∼30–35°C) from the collection site to the laboratory within 5 hours. These samples were used to conduct the experiments within 24 hours after field collection. In the meantime, they were kept at room temperature (∼20–25°C). The absence of virus in all water and mud samples was verified by qRT-PCR prior to use for the experiments. Microbiological and additional physico-chemical parameters were measured in the water samples at the beginning of the experiments; pH and conductivity results demonstrated very few or no differences with the measures made previously in the field (data not shown).

### Aquatic flora and fauna

Freshwater flora and fauna used for the artificial aquatic settings included guppies (*Poecilia reticulata*), Siamese fighting fish (*Betta splendens*), tadpoles (unidentified local species), snails *(Sinotaia quadrata)*, clams (*Corbicula fluminea*), mussels *(Pilsbryoconcha exilis)*, and aquatic plants (*Cabomba caroliniana*). Fish were bought from a private stockbreeder and the other organisms were collected from local rivers or ponds where H5N1 virus circulation was never reported (no permits required).

### Artificial aquatic biotopes: experimental design and settings

**Figure 1:**
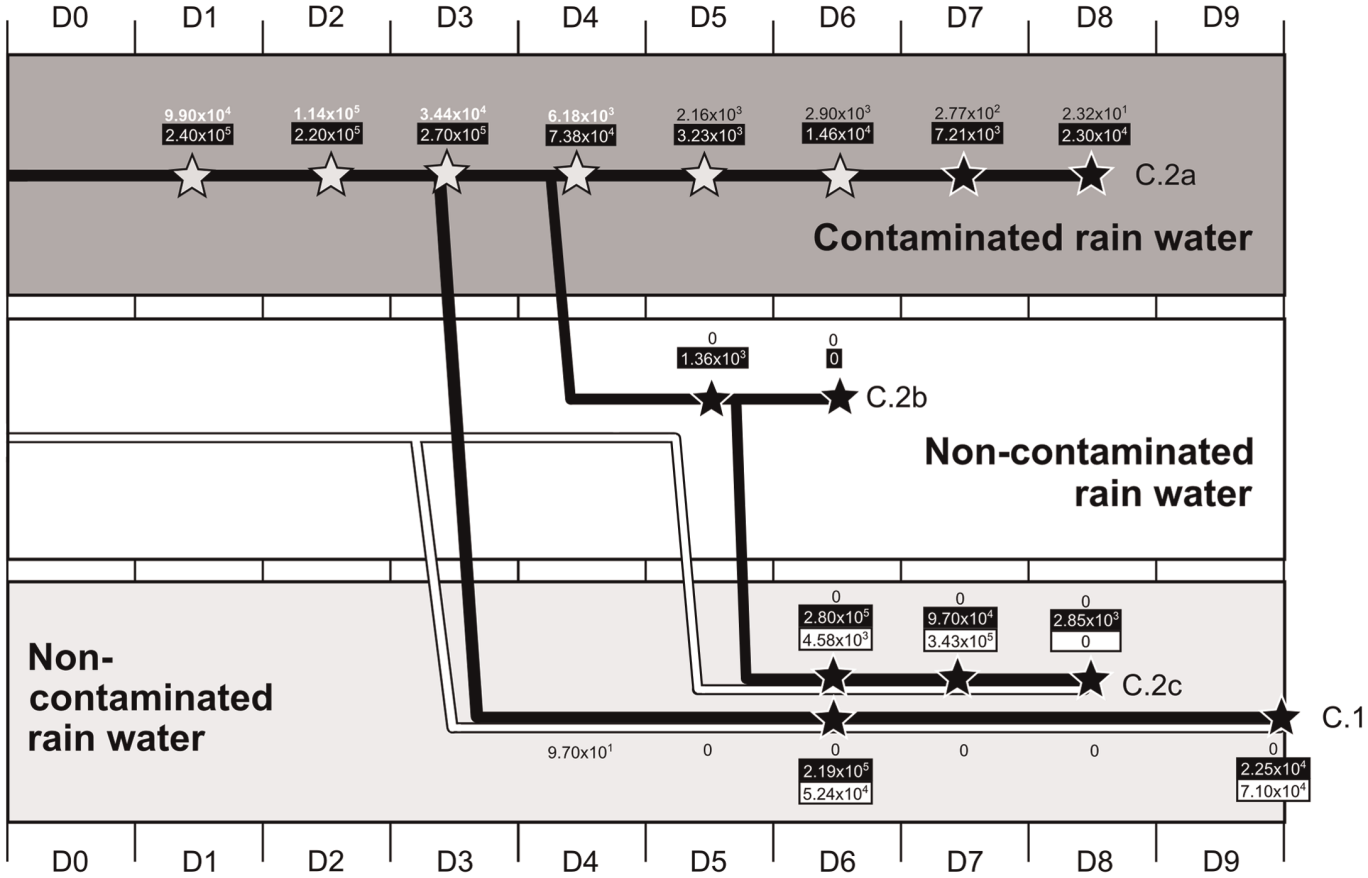
Design of experiments C and laboratory results to assess the role of bivalves (mussels) in the transmission cycle of H5N1 virus in water. The 3 horizontal rectangles represent different types of water in which mussels were immersed: contaminated vs non-contaminated. Black-filled lines represent mussels immersed in contaminated water from day 0 (D0). White-filled lines represent naïve mussels immersed in non-contaminated water from day 0. The two experiments are discernable and identified as C.1 and C.2 (a, b and c). Water samples were collected daily from day 0 (D0) to day 9 (D9). Numbers not included in boxes correspond to viral loads measured in the water samples. When these numbers are displayed in white color, this indicates that infectious virus was also detected. When the numbers are written in black color, this means that the virus was not recovered after inoculation into eggs. Stars indicate collection of mussels' organs for testing. White stars correspond to detection of infectious virus. Black stars indicate that H5N1 virus was not recovered after inoculation into embryonated eggs. Numbers included in black boxes correspond to average viral loads measured in mussels that were immersed in contaminated water. Numbers in white boxes correspond to average viral loads measured in mussels that were immersed in clean water.

**Figure 2:**
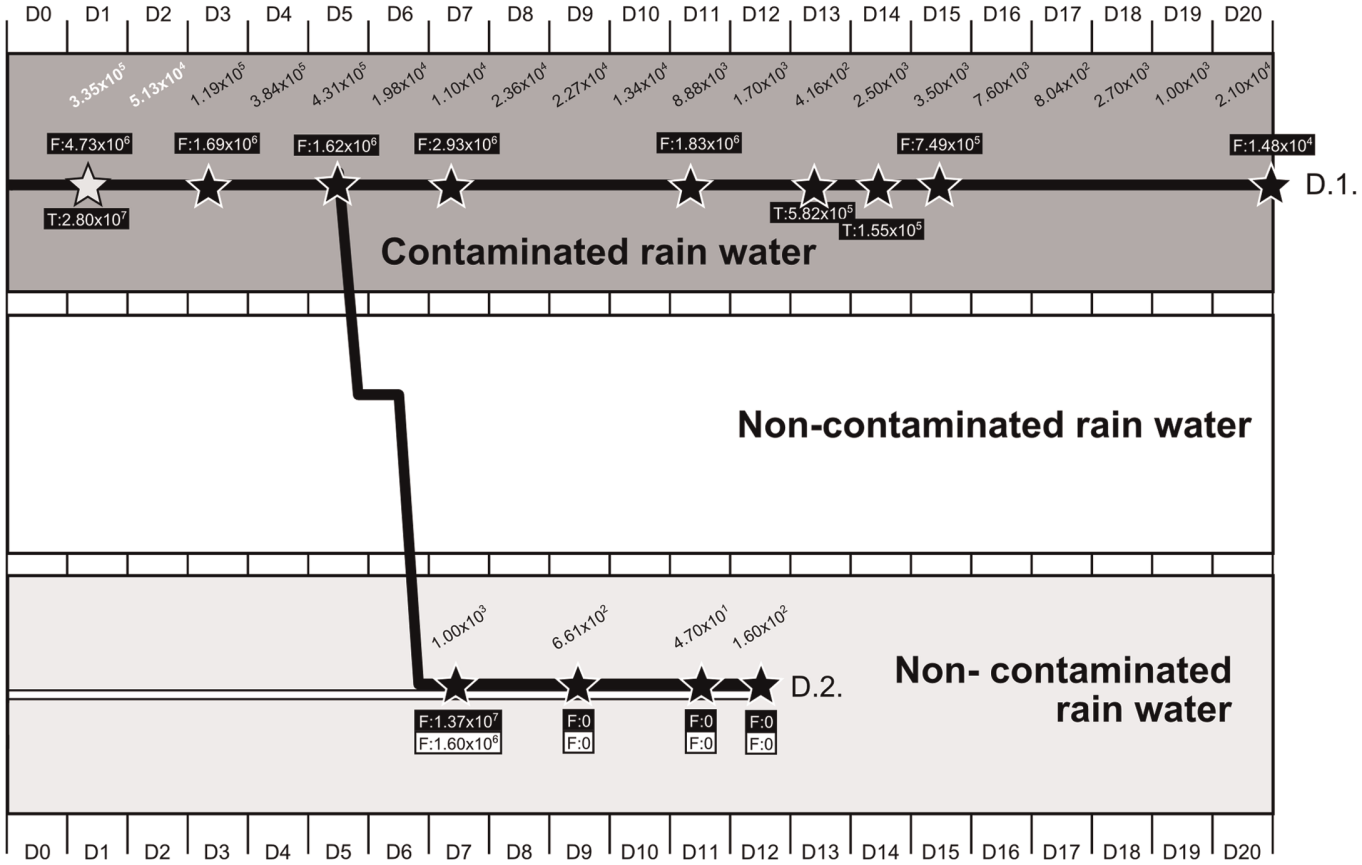
Design of experiments D and laboratory results to assess the role of *Betta splendens* fish in the transmission cycle of H5N1 virus in water. The 3 horizontal rectangles represent different types of water in which animals were immersed: contaminated vs non-contaminated. Tadpoles (T) were immersed along with the male fighting fish (F) in contaminated water. Black-filled lines represent the male fighting fish and the tadpoles immersed in contaminated water from day 0 (D0). At day 5, male contaminated fish were placed over-night in clean water before being exposed to non-contaminated females in clean water. White-filled lines represent naïve female fighting fish immersed in non-contaminated water from day 0. Stars indicate collection of fish and tadpoles (when present) for testing. The two experiments are discernable and identified as D.1 and D.2. White stars correspond to detection of infectious virus in fish. Black stars indicate that H5N1 virus was not recovered from fish's organs after inoculation into embryonated eggs. Numbers included in black boxes correspond to average viral loads measured in fish (F) and tadpoles (T) that were immersed in contaminated water. Numbers in white boxes correspond to average viral loads measured in female fighting fish that were immersed in clean water and exposed to contaminated male fish. Water samples were collected from contaminated water (experiment D.1) from day 0 (D0) up to day 20 (D20). Water samples were collected from non-contaminated water (experiment D.2) from day 0 (D0) to day 12 (D12). The values presented in italic correspond to the viral load measured in water samples. When the numbers in italic are displayed in white color, this indicates that infectious virus was also detected. When the numbers in italic are written in black color, this means that the virus was not recovered after inoculation into eggs.

A total of 4 different series of experiments were carried out. Two series were conducted to investigate the survival and persistence of H5N1 virus in simple biotopes containing only water and mud (A), and in complex biotopes that included aquatic flora and fauna (B) (See Table 1 for details). Two additional series of experiments were set up in order to precisely characterize the role of some aquatic animals in the persistence of H5N1 virus in aquatic environments. The roles of mussels (bivalve molluscs) (C) and endemic *Betta splendens* fish (fighting fish; *Osphronemidae* family) as well as tadpoles (D) were investigated (see Figures 1 and 2 for details).

**Table 1:** Experimental conditions used for the study in simple (A) and complex (B) biotopes.

Series	#		Virus origin^a^	Virus concentration (EID50/mL water)	T°^b^	Water origin	Mud origin	Flora/fauna
**Simple biotopes (A)**	**A.1.**		Avian	5×10^4^	25	Pond 1	NA	No
						Pond 2		
						Rain		
			Human	5×10^4^	25	Pond 1	NA	No
						Pond 2		
						Lake		
						Rain		
	**A.2.**	**A.2.1.**	Avian	5×10^4^	25	Pond 1	Pond 1	No
						Pond 2	Pond 2	
						Lake	Lake	
		**A.2.2.**	Avian	5×10^2^	**22**	Pond 2	Pond 2	No
					25	Pond 1	Pond 1	
					**32**	Pond 2	Pond 2	
					**34**	Pond 1	Pond 1	
			Human	5×10^3^	25	Lake	Lake	No
					**32**			
**Complex biotopes (B)**	**B.1**		Avian	5×10^2^	25	Pond 1	Pond 1	Guppies
								Snails
								Clams
								Plants
				5×10^4^		Lake	Lake	Guppies
								Snails
								Clams
								Mussels
								Plants
			Human	5×10^3^	25	Lake	Lake	Guppies
								Tadpoles
								Plants
				5×10^4^				Guppies
								Clams
								Plants
	**B.2**		Avian	5×10^2^	**22**	Pond 2	Pond 2	Guppies
								Snails
					**32**			Clams
								Plants
					**34**	Pond 1	Pond 1	Guppies
								Snails
								Clams
								Plants
			Human	5×10^3^	**32**	Lake	Lake	Guppies
								Tadpoles
								Plants

a Avian origin strain stands for the A/Chicken/Cambodia/LC1AL/2007 strain. Human origin strain stands for the A/Cambodia/408008/2005 strain.

b T° = Temperature (°C).

Different virus concentrations were tested in this study. Concentrations of 2×10^2^ and 5×10^2^ EID50/mL of water were chosen based on the quantity of virus found in the natural environment in Cambodia during previous field studies [10], [16]. The virus concentration of 5×10^3^ EID50/mL of water was determined based on the estimation of the quantity of virus particles that infected ducks might shed in a pond (number of ducks adjusted to the size of the pond according to field observations) [17]. Finally, a higher dose of virus (5×10^4^ EID50/mL of water) was also tested in order to study the virus persistence in case of higher level of contamination.

Experiments of series A and B lasted 14 days each, and were conducted using different H5N1 strains (A/Chicken/Cambodia/LC1AL/2007 and A/Cambodia/408008/2005), different inoculum doses (yielding final estimated concentrations of 5×10^2^, 5×10^3^ or 5×10^4^ EID50/mL water), and different temperatures reflecting the parameters measured in the field during the transmission season in Cambodia (22, 25, 32 or 34°C). Aquariums with a total capacity of 28 litres (38×38×20 cm) were filled with 20 litres water and 5 kilograms mud each, and then were allowed to settle for 24 hours prior to virus inoculation. Water and mud from various origins were also tested: rain, lake, pond 1, pond 2 (as defined above). When flora (about 100 g) and fauna (30 animals of each species) were included in the experiments (B), collection of samples from each of the species used (2 g of plant, 2 animals of each species) was carried out during the first 3 days, and every 3 days from then on. Fifty millilitres of water and 5 grams of mud samples were collected on a daily basis (Table 1).

Experiments in series C and D lasted from 8 to 20 days depending on the setting chosen. The A/Cambodia/408008/2005 strain was used for experiments C, while experiments D involved the use of the virus A/Chicken/Cambodia/LC1AL/2007. All biotopes created for experiments C and D used rain water only. Aquariums filled with 10 litres of water and 40 mussels each were used for experiments C. In experiments D, series of small aquariums containing 500 mL of water and one male fighting fish in addition to one tadpole were used for experiment D1, while only one fighting fish was included in experiment D2 (Figure 2). In experiments C, the water was maintained at 25°C at all times, while it was kept at ambient BLS-3 laboratory temperatures for experiments D. The temperature measured in the water during experiment D varied from 15.1 to 22.5°C, but usually stayed around 18–20°C with an average temperature of 17.4°C (Figure S1). Final estimated virus concentrations in aquariums varied between 2×10^2^ (D) and 5×10^4^ EID50/mL water (C). Water samples of 50 and 10 mL were collected daily during experiments C and D, respectively (Figures 1 and 2).

For all aquariums, inoculation was carried out on day 0 (D0). After collection, water and mud samples were stored in sterile tubes at −80°C until testing. All aquatic animals were humanely sacrificed, following the Animal Use Protocol defined by the Animal Ethics Committee of Institut Pasteur in Cambodia (permit number: AEC/IPC/004/2008), and subsequently dissected in order to collect the main organs of interest: gills, intestines, fins, scales, brain, and remaining carcass in fish; gills, digestive gland, intestines, and remaining carcass in clams and mussels; gills, intestines, and remaining carcass in tadpoles; all organs in snails. Before dissection, the animals were washed 2 times with sterile distilled water in order to avoid contamination of the organs by the water contained in the aquarium.

Prior to testing, organs were weighed and placed into vials containing 1 mL of viral transport medium (VTM) (sterile solution at pH 7.2–7.4 containing 26.5 g/l of tryptose phosphate broth, 5 g/l of gelatine, 50 mg/l of fungizon, 1 million units/l of penicillin, 1 g/l of streptomycin and 80 mg/l of gentamycin) and stored at −80°C and in 10% formalin solution (prepared from formaldehyde 37% commercial solution diluted in water) stored at room temperature. Plant samples were also weighed and stored in VTM at −80°C until use.

Each experiment conducted was coupled with a control experiment, using the exactly same conditions, but without virus.

### Preparation of samples for RNA extraction and/or virus isolation

#### Virus concentration in water

All water samples were concentrated using the method described by Khalenkov et al. [18], to obtain a final volume of 1 mL of concentrate for each sample. The limit of detection of this technique was 3×10^−2^ EID50/ mL, as previously determined [18].

#### Virus elution and concentration in mud

All mud specimens collected went through an elution step with a 10% beef extract solution at pH 7 followed by a polyethylene glycol-precipitation (PEG) step for virus and RNA concentration, as described previously [19]. The limit of detection of H5N1 virus in mud was 1.6×10^4^ RNA copies/g of mud (approximately 50 TCID50/ g of mud) [20].

Precisely, for each mud sample, 5 grams were eluted in 25 mL of elution buffer (10% beef extract solution). One millilitre of the eluted sample was then kept for a first virus detection, while the remaining volume went through the additional concentration step with PEG so as to obtain a final volume of concentrated sample of 1 mL. Mud eluates and concentrates were then used for RNA extraction and qRT-PCR or virus isolation.

#### Homogenization of animal and vegetal samples

All solid samples (animals' organs, plants) were weighed and kept in 1 mL VTM before undergoing a homogenization step using the MagNa Lyser Instrument (ROCHE, Mannheim, Germany) for 3 runs of 50 seconds at 5000× g. The homogenized samples were then used for further RNA extraction and eventually virus isolation.

### Total nucleic acid extraction and amplification by real-time RT-PCR

All samples processed as described above were mixed with a solution containing a mixture of antibiotics and antifungal drugs prior to RNA extraction or virus isolation, in order to reduce the number of contaminating microorganisms in the samples [18], [19]. MagNa Pure LC Total Nucleic Acid Isolation Kit (Roche Diagnostics) on MagNa Pure LC Instrument (Roche Diagnostics GmbH, Mannheim, Germany) were then used for viral RNA extraction of all eluated/concentrated/homogenized samples (200 µL), following the manufacturers' recommendations. Quantitative real-time RT-PCR (qRT-PCR) targeting the hemagglutinin (H5), matrix (MA) and neuraminidase (N1) genes were performed on all RNA extracted. H5, MA and N1 synthetic RNA were used as internal controls and for quantification. Water and mud samples mixed with H5N1 virus at a concentration higher than the limit of detection were used as positive controls [19]. No positive controls were available for testing the animal and plant specimens.

### Virus isolation in embryonated hen eggs

All samples that tested positive by qRT-PCR were inoculated into 9-to-11-days old SPF embryonated hen eggs. Each specimen was inoculated into 3 eggs. One hundred microlitres were injected into the amniotic cavity and 100 µL into the allantoic cavity. The eggs were then incubated for 48 hours at 37°C and chilled overnight at 4°C. The AAF was then harvested and standard hemagglutination (HA) tests were performed to detect the presence of virus before confirmation by qRT-PCR. HA tests were performed in 96-wells microtiter plates with 0.75% guinea pig red blood cells and serial 2-fold dilutions of AAF. When the HA test was negative, the AAF from each of the three eggs was pooled and inoculated into a second and then a third series of 3 eggs. A maximum of three passages were performed for each sample.

### Histopathology and immunohistochemistry

Immunohistochemical staining of the tissues obtained from the mussels and fish of experiment C and D was carried out for the influenza nucleoprotein using HB65 (European Veterinary Laboratories, Netherlands) as described in previously published reports [21].

## Results

### Survival of infectious particles and persistence of virus RNA in water

**Figure 3:**
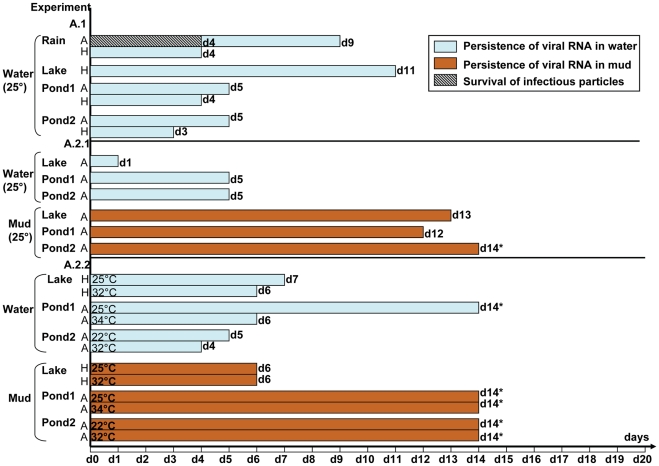
Survival of infectious particles and persistence of virus RNA in simple biotopes (experiments A). A.1: only water of various origins maintained at 25°C and inoculated to a final concentration of 5×10^4^ EID50/mL with H5N1 virus of animal or avian origin. A.2:water and mud containing an estimated final concentration of virus of avian origin of 5×10^4^ EID50/mL water maintained at 25°C (A.2.1) or various concentrations of viruses of avian and human origins and maintained at various temperatures (A.2.2). A: Avian origin strain stands for the A/Chicken/Cambodia/LC1AL/2007 strain. H: Human origin strain stands for the A/Cambodia/408008/2005 strain.*last day of the corresponding experiment at which samples could be collected and tested.

**Figure 4:**
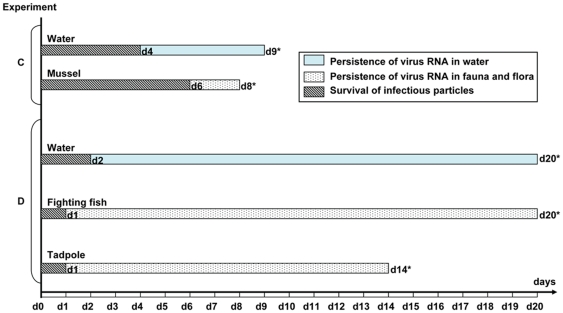
Survival of infectious particles and persistence of virus RNA in water and fauna in experiments C and D. C: water inoculated with the virus of human origin, at a final estimated concentration of 5×10^4^ EID50/mL, maintained at 25°C and containing mussels. D: water inoculated with the virus of avian origin, at a final estimated concentration of 2×10^2^ EID50/mL, maintained at 17°C and containing fighting fish and tadpoles.*last day of the corresponding experiment at which samples could be collected and tested.

Contaminated rain water was the only type of water from which infectious particles could be recovered (Figures 3 and 4; Table S2), although these viruses could not be detected any later than 4 days post-inoculation. In the same experimental conditions, the virus of human origin could not be isolated in embryonated eggs and a trace of its RNA was detected for a shorter period of time than when using the avian isolate (experiment A.1, Figure 3; Table S2). When animals (without plants or mud) were introduced (experiments C with mussels and D with fish and tadpoles) (Figure 4; Table S2), infectious particles of both animal and human origins were then isolated and the RNA persisted for a longer period than in the presence of mud and plants, and at higher levels, especially when the average water temperature was low (17°C).

**Figure 5:**
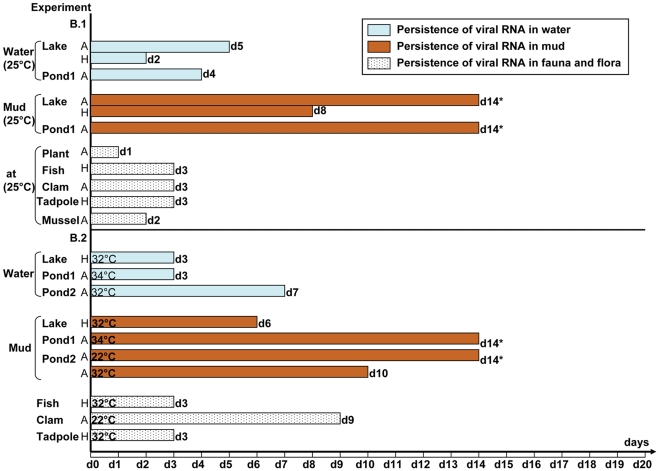
Survival of infectious particles and persistence of virus RNA in complex biotopes (experiments B). B.1: complex biotopes inoculated with virus of avian or human origins at various final concentrations and maintained at 25°C. B.2: complex biotopes inoculated with virus of avian or human origins at various final concentrations and maintained at various temperatures. A: Avian origin strain stands for the A/Chicken/Cambodia/LC1AL/2007 strain. H: Human origin strain stands for the A/Cambodia/408008/2005 strain.*last day of the corresponding experiment at which samples could be collected and tested.

No infectious particle could be isolated from contaminated lake water, although viral RNA was detectable between 1 to 11 days post-inoculation depending on the conditions tested (Figures 3 and 5; Table S2).

No infectious particles could be recovered from pond water. In one instance, viral RNA persisted in water for as long as 14 days (end of the experiment) (experiment A.2.2, Pond 1, Figure 3; Table S2) although at very low titre at this last testing point (52 RNA copies/mL). In all other experiments, viral RNA was detected from contaminated pond water, from 2 to 14 days post-inoculation, with viral loads varying from 2 to 1700 RNA copies/mL.

### Survival of infectious particles and persistence of virus RNA in mud

Infectious particles could not be recovered from any mud samples in any of the experiments conducted in this study (Figure 3 and 5; Table S3). Viral RNA could always be detected by qRT-PCR in mud specimens between 1 to 14 days after inoculation. When only considering the mud samples obtained from the lake, the RNA of the avian strain seemed to persist for longer periods (13–14 days) than the RNA of the human isolate (1–8 days), regardless of the other parameters (experiment B.1, Figure 5; Tables S3 and S7). Globally, in experiments A and B using the avian strain, RNA was detectable for longer periods of time in mud (10 to 14 days) than in water specimens (1 to 7 days), except for one experiment in which viral RNA was still detectable in both water and mud specimens until the very end of the experiment (experiment A.2.2, Figure 3 and 5; Tables S2, S3 and S7). The viral loads measured in the water samples were on an average 3000 times lower than those observed in mud specimens. With the human strain the durations of RNA persistence in water and mud were comparable but the viral loads were approximately 4700 times higher in mud than in water (Table S7).

### Survival of infectious particles and persistence of virus RNA in aquatic flora and fauna

Infectious particles were only isolated from animal organs in experiments C and D, in which mussels, tadpoles and fighting fish were immersed in contaminated rain water in the absence of mud (Figure 4, Table S4). Survival of infectious particles ranged from 1 day in tadpoles and fighting fish (H5N1 strain of avian origin) to 6 days in mussels (with virus of human origin). RNA was detected in tadpoles up to 14 days after immersion in contaminated water (last day of the experiment) and until the end of respectively the 8 and 20 days of experiment in mussel and fighting fish. Viral loads detected in the organs of these animals were relatively high, ranging from 10^2^ to 10^7^ copies per gram (Figure 1 and 2; Tables S4, S5 and S6). The viral loads detected in the different organs of mussels and fish tested did not show any significant tendencies, and thus did not allow us to draw any conclusions regarding possible specific H5N1 tropisms towards certain organs in these aquatic animals, be it for the avian or the human strain (Tables S5 and S6). As for the other aquatic animals and for the plants, only few samples tested positive by RT-PCR (Figure 5; Table S4). Viral RNA persistence varied from 1 to 9 days. RNA could not be detected in the snail species used, nor in any of the animals or plants maintained at a temperature >32°C (Figure 5; Table S4). RNA from the human strain persisted only in guppy fish, whereas RNA from H5N1 virus of avian origin was detected only in clams.

The clams immersed in contaminated water died very quickly, in contrast with those immersed in non-infected water. The presence of mud in lake or pond water (experiments B.1 and B.2) was associated with the absence of infectious particle isolation in tadpoles and with a shorter persistence of virus RNA at a lower viral load (Figure 5; Table S4). In contrast, in the absence of mud, infectious virus was detected on day 1 and the virus RNA persisted for at least 14 days at a high viral load in the animals (experiment D, Figure 4; Tables S4, S6 and S7).

### Transmission of H5N1 virus between bivalve mollucs (experiments C) and between fighting fish (experiments D)

After immersion of mussels in rain water maintained at 25°C and contaminated with the virus A/Cambodia/408008/2005, infectious particles were isolated from water until day 4, from mussels until day 6 and the virus RNA was still detectable in molluscs until the end of the experiment at day 8. Viral loads in mussels varied between 2.40×10^5^ copies on day 1 and 2.30×10^4^ copies per gram of organ on day 8 (experiment C.2, Figure 1).

When transferred into non-contaminated water on day 3 (experiment C.1), RNA was detected in water until day 4 but infectious particles were not found. Infectious virus was not isolated in the infected mussels transferred into clean water on day 6 but the qRT-PCR tested positive until the end of the experiment (experiment C.1., Figure 1). The last viral loads measured were comparable to those of the mussels of the group maintained in contaminated water and interestingly, comparable quantities of RNA were detected in contaminated and exposed molluscs (experiment C.1, Figure 1; Table S5).

When infected mussels were introduced into clean water on day 4 (experiment C.2), no virus could be isolated from the molluscs on day 5, and the water was not contaminated by infectious particles nor by RNA. However, RNA was still detected in infected mussels until day 8 (end of the experiments) and until day 7 in mussels exposed to infected ones (experiment C.2, Figure 1; Table S5).

During the experiment D, fighting fish and tadpoles were immersed in contaminated rain water. The virus was isolated from animal organs until day 1 in both species and in water until day 2. RNA was detected in tadpoles until day 14 (last tadpole tested) and until day 20 (end of the experiment) in both water and fish. Viral loads varied from 2.8×10^7^ copies on day 1 to 1.55×10^5^ copies per gram of organ on day 14 for tadpoles, and from 4.73×10^6^ copies to 1.48×10^4^ copies per gram of organ on day 20 for fighting fish (experiment D.1, Figure 2). When contaminated fighting fish were placed into clean water on day 6, no infectious virus could be isolated from fish or from water. RNA persisted in water until the end of the experiment on day 12 (viral load : 1.60×10^2^ copies/mL) but in fish only until D7, in both infected and exposed animals with 1 log difference in viral loads measured in their organs (experiment D.2, Figure 2).

The viral loads presented here referred to the highest individual values found when analyzing the different organs in each animal group (Tables S5 and S6). In guppy fish, the highest mean viral load was measured in gills (9.1×10^5^ copies/g) followed by fins (4.8×10^5^ copies/g), while the values obtained in the other organs varied from 4.5×10^4^ to 2.8×10^5^ copies/g. In contaminated fighting fish, the highest mean viral loads were observed in gills (1.05×10^6^ copies/g) and brain (4.11×10^5^ copies/g), while the viral loads measured in the other organs collected varied from 1.90×10^3^ to 2.93×10^6^ copies/g (Table S6). In tadpoles, the viral loads were quite similar in all organs (between 1.79 and 2.80×10^7^ copies/g for tadpole immersed during 1 day in infected water and between 9.83×10^4^ and 5.82×10^5^ copies/g for tadpole kept 13–14 days in contaminated water) (Table S6). In clams, viral loads varied between 3.8×10^3^ and 6.28×10^4^ copies/g depending on the organ tested.

### Histopathology and immunohistochemistry

Immunohistochemical staining of the tissues did not confirm the presence of the H5N1 virus antigen in any organ of the mussels and fish tested following experiments C and D.

## Discussion

This study aimed to recreate simple as well as complex aquatic environments with parameters (pH, temperature, salinity, microorganisms, flora, fauna, etc.) as close as possible to those observed in Cambodia, where H5N1 outbreaks are regularly reported, and to observe the survival of the HPAI H5N1 virus in all the different compartments of these artificial aquatic biotopes which have been suggested to be at the origin of asymptomatic or sub-clinical human infections [4], [22]. Infected ducks can shed a large number of virus particles in their faeces but also in saliva and nasal discharge which can therefore easily lead to water contamination [23]. The survival of avian influenza viruses in natural or artificial environments has already been studied in several occasions and a recent review of Stallknecht and Brown commented that the persistence of HPAI H5N1 virus in the environment was still poorly explored [8].

In our experiments, infectious HPAI H5N1 virus could be recovered from water during a maximum of 4 days post-contamination at 25°C but only in rain water. This temperature is commonly observed all year long in Cambodian ponds and lakes, around 20−40 cm beneath the surface, as opposed to the surface where the temperature can easily exceed 30°C. The survival of AIVs in water is known to be shorter when temperature increases [8]. Interestingly, in similar conditions, infectious particles could not be isolated from any of the natural surface water specimens tested (ponds and lake). The pH values measured in this study varied between 7.45 and 8 which were described to be the optimal conditions to maintain the AIVs infectivity [8]. The main physicochemical and microbiological parameters which differed between rain and pond/lake water specimens were: a total absence of chemical oxygen demand (parameter used to indirectly evaluate the organic compounds) with globally lower concentrations of nitrite and nitrate, a higher concentration of sodium and a globally less abundant bacteriological flora in rain water (Table S1). Nazir et al. examined the survival of low pathogenic avian influenza (LPAI) strains and reported that at 20–30°C, the persistence of the viruses was longest in distilled water, second longest in normal saline solution and shortest in surface water [24]. Others demonstrated that the presence of living microorganisms in some waters reduced AIV survival [5], [23]–[25]. These data are in line with our observations which suggest that at the temperature naturally observed in tropical countries like Cambodia, the presence of organic contaminants and microorganisms in natural surface waters are strongly affecting the H5N1 virus survival in water. Additionally, although our samples underwent standard bacteriological analyses, water specimens could have contained a whole range of other microorganisms, including fungi and other microbes, which have not been investigated and which could have potentially been interacting in some unknown way with influenza virus particles. Clean water, which can be found in wells, in some containers, in puddles, etc., are in contrast favourable to the H5N1 virus survival and this seems not to be depending on the initial concentration of the virus i.e. the level of virus contamination.

Interestingly, although experiments were conducted under identical conditions, the H5N1 virus obtained from a human case did not survive in rain water in the absence of fauna. Other authors reported that in experiments where only pH, salinity and temperatures varied, H5N1 viruses appeared to persist for shorter periods than other avian influenza viruses tested [11]. This demonstrated an inter-subtype variation of virus tenacity in water but our results also suggest the existence of an important intra-subtype variation that could be explained by biological variations resulting from differing replication abilities in different hosts, or by yet unknown genetic mutations associated with virus survival in abiotic environments.

The detection of virus RNA by qRT-PCR did not correlate with the recovery of infectious particles. Indeed, in some experiments, infectious virus could not be isolated while RNA could be detected for several days. In the absence of mud, plants or animals, RNA was detected for periods as long as 11 days at 25°C. In complex biotopes, an increase of the temperature from 25°C to 32°C or 34°C reduced the persistence of the RNA (experiments A2.2, Figure 3; Table S2). This is not surprising as RNA is known to be heat labile.

Infectious particles were never isolated in mud specimens although the method used was proven to be efficient [19]. LPAI viruses were reported to survive between 2 and 4 days at temperatures ranging from 20 to 30°C in some lake sediments [26]. The nature of the soil in Cambodia or the biological characteristics of the HPAI H5N1 virus may explain why the viruses did not survive in our mud specimens. It has been described that avian influenza viruses are relatively unstable in the environment due to their lipid envelopes readily being inactivated by several physical factors, organic solvents, and detergents [23]. However, this low detection of infectious particles may also be related to detection limits. For instance, adsorption of live virus on soil micro-particles, or contamination of the samples with environmental bacteria, fungi, or other microorganisms despite prior treatment, could prevent the growth of the virus in hen egg cultures [18], [27]–[30]. As in water, the persistence of the avian H5N1 RNA tends to last longer than that of the human H5N1 strain in the mud, possibly for the same reasons as suggested above. Moreover, RNA persisted for longer periods in mud than in water. Previous publications supported the idea that AIVs could survive for longer in lake sediments than in lake water [26] and that lake and pond sediments could act as a reservoir of influenza viruses [31]. Our experiments cannot lead to similar conclusions as we did not isolate infectious particles from mud but mud and sediments may be preventing RNA from decay within the nucleoprotein, thus allowing it to be detected by qRT-PCR [31]–[33] even though PCR inhibitors are expected to decrease the detection rate of viral RNA in mud. Indeed, in our experiments, such inhibitors were detected in 50% of the soil and mud samples collected from the natural environment in Cambodia (Institut Pasteur in Cambodia, unpublished data). Several authors demonstrated that virus detection in environmental samples could indeed be strongly influenced by many substances present in environmental samples, such as bentonite clay, humic acid or mussel tissue, that can inhibit RT-PCR [34].

The detection thresholds of the assays could also be questioned but the quantity of virus inoculated at the beginning of the experiments should have ended in theoretical concentrations in water and mud above the limit of detection of these methods. Nevertheless, as we did not perform back-titrations immediately after virus inoculation in water, we cannot dismiss the possibility that the starting concentrations were lower than those calculated by simply applying a dilution factor. The initial virus titers used may appear low but they were comparable to those observed in the field during environmental investigation following outbreak in poultry in Cambodian farms [10], [16]. Indeed, one of the main objective of these experiments was to study the persistence of H5N1 virus in conditions as close as possible to the field. The low virus isolation rate could be partially explained by a non-uniform distribution of the virus in the aquarium, although we tried to limit this bias by gently homogenizing the water in the aquarium with a long pipette and by collecting each sample at 4–5 different locations.

To our knowledge, data related to the infection of aquatic animals by AIVs in general is very rare and we did not find any study evaluating interactions between H5N1 virus and aquatic plants either.

The plants maintained in H5N1- infected water in conditions meant to simulate natural ones in Cambodia did not show any contamination by the virus regardless of the virus type and of the virus concentration except for one plant specimen in which viral RNA was detected during the first day of the experiment. This finding is in agreement with the investigation conducted on natural environmental samples collected after an H5N1 outbreak in Cambodia in 2006, which assessed the presence of viral RNA in plants from which no live virus particles were recovered [10]. This suggests that aquatic plants may not help virus survival, nor act as physical support for viral particles dispersed in water.

When molluscs, fish or tadpoles were introduced in aquariums containing rain water, infectious particles of the strain of human origin could subsequently be recovered and the RNA persistence of both human and avian strains increased significantly (up to 20 days in one experiment). This suggests a probable impact of these aquatic animals on the biological cycle of H5N1 virus. Faust et al. highlighted in 2009 the role of clams (*Corbicula fluminea*) in removing - by filtration - the virus from the water, and in reducing the infectivity of LPAI virus [13]. Another study reported that zebra mussels (*Dreissena polymorpha*) were able to accumulate LPAI virus from the surrounding water, and to retain the virus in their bodies over an extended period of time before releasing the virus back into freshwater [14]. As shown on Figure 1, the viral RNA persisted in water until the last day of the experiment (day 8) even in the presence of mussels. We observed a decrease of the viral load measured in water (3 logs in 8 days) but also in mussels (1 log in 8 day). In experiment C, infectious particles were detected in water during 4 days, and during 6 days in mussels (Figure 1). Once transferred to clean water, the infectious particles disappeared from the mussels and did not contaminate the water. Virus RNA was detected for few days in infected and exposed mussels. It seems that the species of mussel used in our experiments did not favor the detection of infectious H5N1 virus in water (by comparison with water alone). The animals probably filtered and concentrated the RNA to some extent but also probably released some nucleic acids since the initially clean water was slightly contaminated afterwards, and that virus RNA was detected in mussels exposed to infected ones. Our experiments suggest that mussels may be able to at least release nucleic acids in the environment. If they released some infectious virus, it was below the detection threshold of our technique.

Histopathology suggested that the virus was not replicating in mussels and thus that the detection of infectious particles in these molluscs was probably only the result of their natural capacity to filter water. However, this observation may also only be the result of the lower sensitivity of the immunohistochemical method compared to qRT-PCR. Fish (*Betta splendens*) and tadpoles carried detectable HPAI H5N1 virus particles for 1 day only, while infectious particles were isolated from the seeded water until day 2. Interestingly, fish and tadpoles as well as water specimens tested positive by qRT-PCR for 20 days while RNA persisted for a maximum length of 9 days in aquariums containing only rain water (experiment A.1). After 1 day of transit of the infected fish in clean water, the RNA was transmitted to exposed fish for only a short period of time. But surprisingly, the initially non-contaminated water tested positive by qRT-PCR for an additional 5 days. The histopathology analyses did not show the presence of virus antigen in the animal tissues tested, suggesting that the virus was not replicating in these tissues. Nevertheless, because of the limited sensitivity of this method, this result should be interpreted with caution. Fish and tadpoles seemed to be able to concentrate the RNA but not the infectious virus in their organs, and to efficiently protect this RNA from decay. These animals also released nucleic acids in water, allowing the detection of H5N1 virus by qRT-PCR for longer periods. Their gills probably acted as filtration systems while the RNA detected in their intestines was probably only the result of the passage of the RNA through the digestive tract, presumably together with food. The detection of nucleic acids but not of infectious virus in fish's organ tissues, including in brain, could be the result of a contamination with nucleic acids from contaminated water during the delicate dissection of the tiny animals, although the animals were all washed in sterile distilled water before the dissection.

As often demonstrated through the years, aquatic waterfowl such as ducks, when infected, can shed large amount of virus in their feces, saliva and nasal discharge, all of these potentially resulting in environmental contamination [23], [35]. Indeed, in several instances, environmental surfaces, including water, were found to be contaminated by HPAI H5N1 virus during or after outbreaks in poultry [9], [10], [17], [33], [35]. Thus, as shown in our study in waters heavily contaminated by the virus, aquatic molluscs or fish could be passive carriers of avian influenza H5N1 virus and may potentially contaminate domestic or wild birds but also human hosts if correct cleaning and cooking conditions are not applied prior to consumption.

It should be noted that even though this study was meant to reproduce as faithfully as possible the real field conditions, our experiments differed from those by many elements, including the nature of the inoculum. While most environmental materials are contaminated by faeces, saliva, or other organic secretions, our inoculum was amnio-allantoic fluid. In the field, however, it is noteworthy that influenza viruses are protected by organic materials such as nasal secretions or faeces, which may increase their resistance to physical and chemical inactivation [23].

Although in our experimental study HPAI H5N1 infectious virus could not be detected in environmental water and mud from pond and lake origins, we cannot exclude the possibility that the virus could survive in different areas where physico-chemical and microbiological parameters could differ. Indeed, previous studies suggested that even minor fluctuations in temperature, pH and salinity at levels normally encountered in natural aquatic habitats may enhance or diminish environmental persistence [8]. In addition, we may not have selected for our experiments the strains that had the best fitness to persist in the environment. The persistence of viral RNA for periods of 2 weeks in environmental materials is an indicator that at some time, even for a short period, infectious particles were present. Thus, we can not rule out the risk of human contamination from the environment, especially since this risk was suggested and reported in several investigations [4], [6], [9], [22], [36]–[38]. A contaminated environment could provide a continuing source of virus, and restricted access of human and animals to potentially contaminated ponds and lakes should be recommended during and after outbreaks in addition to information regarding the potential risk encountered during collection and consumption of aquatic molluscs or fish. In particular, bathing or swimming activities in contaminated ponds in Cambodia have been clearly identified as a risk factor for human contamination by H5N1 virus [4], [22]. Additional factors explaining survival and persistence of HPAI viruses in the environment are still to be elucidated, but our results, along with previous data, support the idea that environmental surveillance is of major relevance for avian influenza control programs.

## Supporting Information

Figure S1 **Water temperature measured during experiments D.**
(TIF)

Table S1 **Physico-chemical and microbiological parameters measured in water samples prior to experimental contamination.**
(RTF)

Table S2 **Survival of infectious particles and persistence of virus RNA in water specimens of various origins.**
(DOC)

Table S3 **Survival of infectious particles and persistence of virus RNA in mud specimens of various origins.**
(DOC)

Table S4 **Survival of infectious particles and persistence of virus RNA in presence of aquatic flora and fauna.**
(DOC)

Table S5 **Viral load (number of H5 RNA copies/g) measured in different mussel organs obtained in experiment C.2 (virus A/Cambodia/408008/2005).**
(DOC)

Table S6 **Viral load (number of H5 RNA copies/g) in different fish and tadpole organs obtained from experiments D (virus A/chicken/Cambodia/LC1AL/2007).**
(DOC)

Table S7 **Survival of infectious particles and persistence of virus RNA in aquatic environments: compiled data.**
(RTF)
