# Supplementary figures and images for: Highly Pathogenic Influenza A(H5N1) Virus Survival in Complex Artificial Aquatic Biotopes
Source: PLoS One. 2012 Apr 13;7(4):e34160. doi: 10.1371/journal.pone.0034160 (PMC3325971; doi:10.1371/journal.pone.0034160)

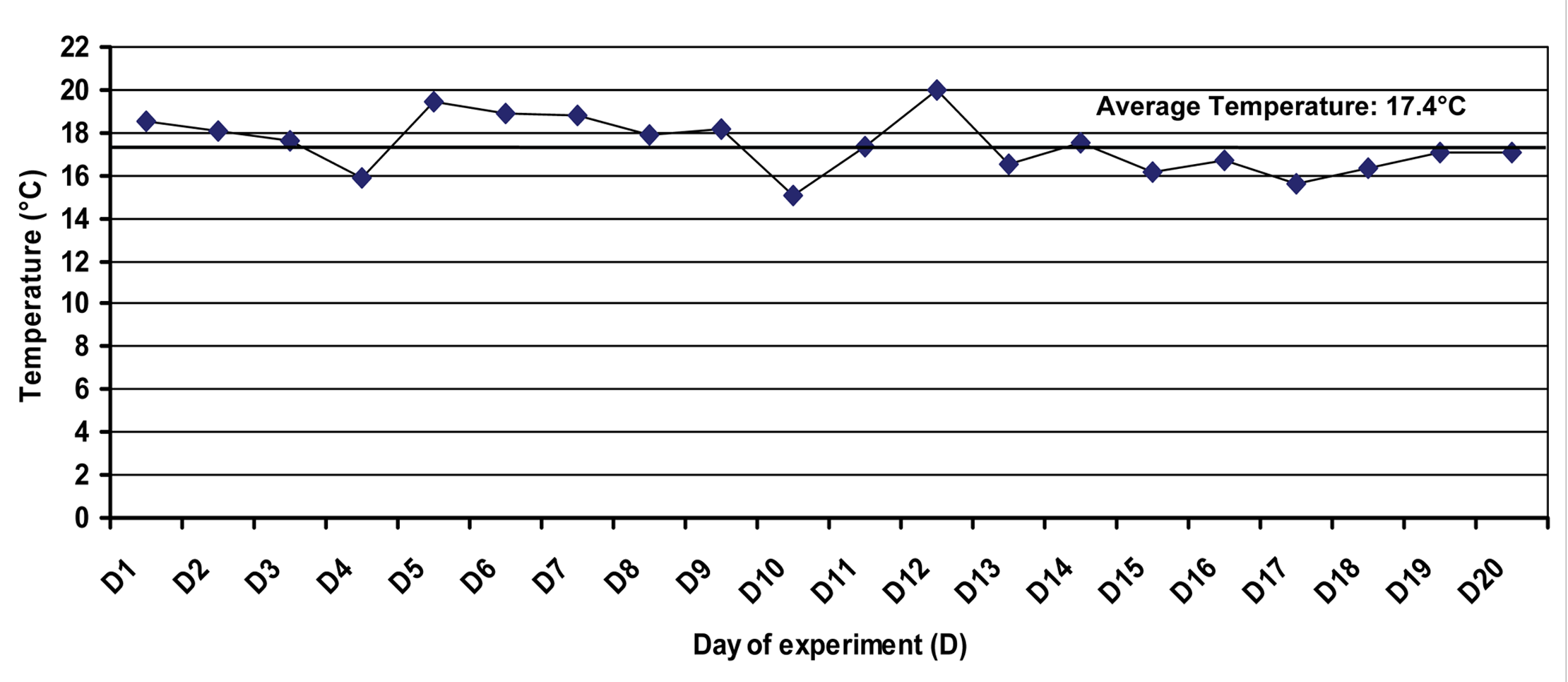

Supplement: Figure S1 — Water temperature measured during experiments D. (TIF) [file pone.0034160.s001.tif]
