# Supplementary material for: Highly Pathogenic Influenza A(H5N1) Virus Survival in Complex Artificial Aquatic Biotopes
Source: PLoS One. 2012 Apr 13;7(4):e34160. doi: 10.1371/journal.pone.0034160 (PMC3325971; doi:10.1371/journal.pone.0034160)
Supplement: Table S1 — Physico-chemical and microbiological parameters measured in water samples prior to experimental contamination. (RTF) [file pone.0034160.s002.rtf]

Supplementary Table 1: Physico-chemical and microbiological parameters measured on water samples prior to experimental contamination.

		Water origin	
		Pond 1	Pond 2	Lake	Rain	
Parameters measured on collection sites	Temperature (°C)	34	31.7	33.4	18.5	
	Conductivity (µS)	60	260	154	/	
Physico-chemical analysis in laboratory on samples collected and strored	Turbidity (NTU)	112	6	35	6	
	pH	7.50	7.45	7.75	8	
	Chloride (mg/L)	10	40	63	42	
	Ammonium (mg/L)	0.23	0.25	0.04	0.15	
	Nitrite (mg/L)	0.06	0.16	0.14	0	
	Nitrate (mg/L)	0.34	4.28	1.94	0.5	
	Hardness (mg/L)	50	85	95	125	
	Total Chlorine (mg/L)	0.08	0.05	0.06	0.05	
	Iron (mg/L)	0	0	0.04	0.09	
	Total Nitrogen (mg/L)	0	2.2	1.6	1.9	
	Sulphate (mg/L)	0	5	6	4	
	Sodium (mg/L)	0	4	3	21	
	Chemical Oxygen Demand (mg/L)	372	350	13	0	
	Phosphate (mg/L)	0	0	2.4	2.2	
Bacteriological analysis	Total aerobic plate count at 37°C-24h (CFU/mL)	+++ (uncountable)	580	+++ (uncountable)	400	
	Total aerobic plate count at 22°C-72h (CFU/mL)	+++ (uncountable)	852	+++ (uncountable)	8600	
	Total Coliforms (CFU/100mL)	2.9104	356	3104	3000	
	Thermotolerant Coliforms (CFU/100mL)	2.2104	278	4.4103	2700	
	Enterococcus faecalis (CFU/100mL)	0	32	20	260	
	Sulphite reducing anaerobies (CFU/20mL)	200	48	40	<1	
