# Supplementary material for: Highly Pathogenic Influenza A(H5N1) Virus Survival in Complex Artificial Aquatic Biotopes
Source: PLoS One. 2012 Apr 13;7(4):e34160. doi: 10.1371/journal.pone.0034160 (PMC3325971; doi:10.1371/journal.pone.0034160)
Supplement: Table S2 — Survival of infectious particles and persistence of virus RNA in water specimens of various origins. (DOC) [file pone.0034160.s003.doc]

**Supplementary Table 2. Survival of infectious particles and persistence of virus RNA** in water specimens of various origins.

| **Water origin** | **Series #^a^** | **Virus origin^b^** | **Virus concentration (EID50/mL water)** | **T°** | **Mud** | **Flora/fauna** | **Survival of infectious particles in water (days)** | **Persistence of viral RNA in water (days)** | **N# viral RNA copies /mL of water** |
| --- | --- | --- | --- | --- | --- | --- | --- | --- | --- |
| **Rain** | **A.1** | Avian | 5×10^4^ | 25 | No | No | **4** | **9** | **1.30×10^1^** |
|  |  | Human | 5×10^4^ | 25 | No | No | 0 | **4** | **4.20×10^0^** |
|  | **C** | Human | 5×10^4^ | 25 | No | **Yes** | **4** | **9*** | **5.50×10^2^** |
|  | **D** | Avian | **2×10^2^** | **17** | No | **Yes** | **2** | **20*** | **2.10×10^4^** |
| **Lake** | **A.1** | Human | 5×10^4^ | 25 | No | No | 0 | **11** | **2.22×10^2^** |
|  | **A.2.1** | Avian | 5×10^4^ | 25 | **Yes** | No | 0 | **1** | **1.55×10^2^** |
|  | **A.2.2** | Human | **5×10^3^** | 25 | **Yes** | No | 0 | **7** | **3.22×10^0^** |
|  |  |  |  | **32** | **Yes** | No | 0 | **6** | **1.05×10^1^** |
|  | **B.1** | Avian | 5×10^4^ | 25 | **Yes** | **Yes** | 0 | **5** | **1.56×10^1^** |
|  |  | Human | **5×10^3^** | 25 | **Yes** | **Yes** | 0 | **7** | **9.02×10^0^** |
|  |  |  | 5×10^4^ | 25 | **Yes** | **Yes** | 0 | **2** | **4.75×10^1^** |
|  | **B.2** | Human | **5×10^3^** | **32** | **Yes** | **Yes** | 0 | **3** | **1.09×10^1^** |
| **Pond 1** | **A.1** | Avian | 5×10^4^ | 25 | No | No | 0 | **5** | **2.00×10^0^** |
|  |  | Human | 5×10^4^ | 25 | No | No | 0 | **4** | **2.22×10^0^** |
|  | **A.2.1** | Avian | 5×10^4^ | 25 | **Yes** | No | 0 | **5** | **3.50×10^0^** |
|  | **A.2.2** | Avian | **5×10^2^** | 25 | **Yes** | No | 0 | **14*** | **5.20×10^1^** |
|  |  |  |  | **34** | **Yes** | No | 0 | **6** | **2.02×10^2^** |
|  | **B.1** | Avian | **5×10^2^** | 25 | **Yes** | **Yes** | 0 | **4** | **1.28×10^1^** |
|  | **B.2** | Avian | **5×10^2^** | **34** | **Yes** | **Yes** | 0 | **3** | **1.11×10^1^** |
| **Pond 2** | **A.1** | Avian | 5×10^4^ | 25 | No | No | 0 | **5** | **8.00×10^0^** |
|  |  | Human | 5×10^4^ | 25 | No | No | 0 | **3** | **3.96×10^1^** |
|  | **A.2.1** | Avian | 5×10^4^ | 25 | **Yes** | No | 0 | **5** | **9.16×10^1^** |
|  | **A.2.2** | Avian | **5×10^2^** | **22** | **Yes** | No | 0 | **5** | **1.82×10^2^** |
|  |  |  |  | **32** | **Yes** | No | 0 | **4** | **1.78×10^1^** |
|  | **B.2** | Avian | **5×10^2^** | **22** | **Yes** | **Yes** | 0 | **2** | **1.70×10^3^** |
|  |  |  |  | **32** | **Yes** | **Yes** | 0 | **7** | **7.88×10^2^** |

^a^ Series numbers as defined in Table 1. A = Simple biotopes, with A.1 = only water, no mud, A.2 = water and mud at 25°C with the standard inoculum dose of 5×10^4^ EID50/mL water (A.2.1), and at various temperatures with different inoculum doses (A.2.2). B = Complex biotopes including the presence of flora/fauna, at 25°C (B.1) and other temperatures (B.2).

^b^ Avian strain stands for the A/Chicken/Cambodia/LC1AL/2007 strain. Human strain stands for the A/Cambodia/408008/2005 strain.

T° = Temperature (°C).

*last day of the corresponding experiment at which samples could be collected and tested.
