# Supplementary material for: Highly Pathogenic Influenza A(H5N1) Virus Survival in Complex Artificial Aquatic Biotopes
Source: PLoS One. 2012 Apr 13;7(4):e34160. doi: 10.1371/journal.pone.0034160 (PMC3325971; doi:10.1371/journal.pone.0034160)
Supplement: Table S3 — Survival of infectious particles and persistence of virus RNA in mud specimens of various origins. (DOC) [file pone.0034160.s004.doc]

**Supplementary Table 3. Survival of infectious particles and persistence of virus RNA in mud specimens of various origins.**

| **Mud origin** | **Series #^a^** | **Virus origin^b^** | **Virus concentration (EID50/mL water)** | **T°** | **Flora/fauna** | **Survival of infectious particles in mud (days)** | **Persistence of viral RNA in mud (days)** | **N# viral RNA copies /g of mud** |
| --- | --- | --- | --- | --- | --- | --- | --- | --- |
| **Lake** | **A.2.1** | Avian | 5×10^4^ | 25 | No | 0 | **13** | **1.26×10^4^** |
|  | **A.2.2** | Human | **5×10^3^** | 25 | No | 0 | **6** | **1.20×10^3^** |
|  |  |  |  | **32** | No | 0 | **6** | **4.30×10^3^** |
|  | **B.1** | Avian | 5×10^4^ | 25 | **Yes** | 0 | **14*** | **1.30×10^4^** |
|  |  | Human | **5×10^3^** | 25 | **Yes** | 0 | **8** | **1.09×10^3^** |
|  |  |  | 5×10^4^ | 25 | **Yes** | 0 | **1** | **1.60×10^4^** |
|  | **B.2** | Human | **5×10^3^** | **32** | **Yes** | 0 | **6** | **1.13×10^3^** |
| **Pond 1** | **A.2.1** | Avian | 5×10^4^ | 25 | No | 0 | **12** | **1.26×10^2^** |
|  | **A.2.2** | Avian | **5×10^2^** | 25 | No | 0 | **14*** | **2.23×10^2^** |
|  |  |  |  | **34** | No | 0 | **14*** | **1.53×10^2^** |
|  | **B.1** | Avian | **5×10^2^** | 25 | **Yes** | 0 | **14*** | **2.60×10^2^** |
|  | **B.2** | Avian | **5×10^2^** | **34** | **Yes** | 0 | **14*** | **4.45×10^2^** |
| **Pond 2** | **A.2.1** | Avian | 5×10^4^ | 25 | No | 0 | **14*** | **2.77×10^3^** |
|  | **A.2.2** | Avian | **5×10^2^** | **22** | No | 0 | **14*** | **1.79×10^3^** |
|  |  |  |  | **32** | No | 0 | **14*** | **5.50×10^1^** |
|  | **B.2** | Avian | **5×10^2^** | **22** | **Yes** | 0 | **14*** | **3.05×10^2^** |
|  |  |  |  | **32** | **Yes** | 0 | **10** | **1.29×10^2^** |

^a^ Series numbers as defined in Table 1. A = Simple biotopes, with A.1 = only water, no mud, A.2 = water and mud at 25°C with the standard inoculum dose of 5×10^4^ EID50/mL water (A.2.1), and at various temperatures with different inoculum doses (A.2.2). B = Complex biotopes including the presence of flora/fauna, at 25°C (B.1) and other temperatures (B.2).

^b^ Avian strain stands for the A/Chicken/Cambodia/LC1AL/2007 strain. Human strain stands for the A/Cambodia/408008/2005 strain.

T° = Temperature (°C).

*last day of the corresponding experiment at which samples could be collected and tested.
