# Supplementary material for: Highly Pathogenic Influenza A(H5N1) Virus Survival in Complex Artificial Aquatic Biotopes
Source: PLoS One. 2012 Apr 13;7(4):e34160. doi: 10.1371/journal.pone.0034160 (PMC3325971; doi:10.1371/journal.pone.0034160)
Supplement: Table S4 — Survival of infectious particles and persistence of virus RNA in presence of aquatic flora and fauna. (DOC) [file pone.0034160.s005.doc]

**Supplementary Table 4. Survival of infectious particles and persistence of virus RNA** in presence of aquatic flora and fauna.

| **Flora/fauna** | **Series #^a^** | **Virus origin^b^** | **Virus concentration (EID50/mL water)** | **T°** | **survival of infectious particles in flora/fauna (days)** | **Persistence of viral RNA in flora/fauna (days)** | **N# viral RNA copies/g (organs/plant)** |
| --- | --- | --- | --- | --- | --- | --- | --- |
| **Plants** | **B.1** | Avian | 5×10^2^ | 25 | 0 | 0 |  |
|  |  |  | 5×10^4^ | 25 | 0 | **1** | **1.20×10^4^** |
|  |  | Human | 5×10^3^ | 25 | 0 | 0 | 0 |
|  |  |  | 5×10^4^ | 25 | 0 | 0 | 0 |
|  | **B.2** | Avian | 5×10^2^ | **22** | 0 | 0 | 0 |
|  |  |  |  | **32** | 0 | 0 | 0 |
|  |  |  |  | **34** | 0 | 0 | 0 |
|  |  | Human | 5×10^4^ | 25 | 0 | 0 | 0 |
| **Guppies** | **B.1** | Avian | 5×10^2^ | 25 | 0 | 0 | 0 |
|  |  |  | 5×10^4^ | 25 | 0 | 0 | 0 |
|  |  | Human | 5×10^3^ | 25 | 0 | **3** | **9.33×10^3^** |
|  |  |  | 5×10^4^ | 25 | 0 | 0 |  |
|  | **B.2** | Avian | 5×10^2^ | **22** | 0 | 0 |  |
|  |  |  |  | **32** | 0 | 0 |  |
|  |  |  |  | **34** | 0 | 0 |  |
|  |  | Human | 5×10^3^ | **32** | 0 | **3** | **5.29×10^4^** |
| **Snails** | **B.1** | Avian | 5×10^2^ | 25 | 0 | 0 |  |
|  |  |  | 5×10^4^ | 25 | 0 | 0 |  |
|  | **B.2** | Avian | 5×10^2^ | **22** | 0 | 0 |  |
|  |  |  |  | **32** | 0 | 0 |  |
|  |  |  |  | **34** | 0 | 0 |  |
| **Clams** | **B.1** | Avian | 5×10^2^ | 25 | 0 | 0 |  |
|  |  |  | 5×10^4^ | 25 | 0 | **3*** | **5.70×10^4^** |
|  |  | Human | 5×10^4^ | 25 | 0 | 0 |  |
|  | **B.2** | Avian | 5×10^2^ | **22** | 0 | **9*** | **9.08×10^2^** |
|  |  |  |  | **32** | 0 | 0 |  |
|  |  |  |  | **34** | 0 | 0 |  |
| **Tadpoles** | **B.1** | Human | 5×10^3^ | 25 | 0 | **3** | **8.08×10^3^** |
|  | **B.2** | Human | 5×10^3^ | **32** | 0 | **3** | **1.59×10^4^** |
|  | **D.1** | Avian | 2×10^2^ | **17** | **1** | **14*** | **1.12×10^6^** |
| **Mussels** | **B.1** | Avian | 5×10^4^ | 25 | 0 | **2** | **3.30×10^3^** |
|  | **C** | Human | 5×10^4^ | 25 | **6** | **8*** | **2.25×10^4^** |
| **Fighting fish** | **D** | Avian | 2×10^2^ | **17** | **1** | **20*** | **1.48×10^4^** |

^a^ Series numbers as defined in Table 1. A = Simple biotopes, with A.1 = only water, no mud, A.2 = water and mud at 25°C with the standard inoculum dose of 5×10^4^ EID50/mL water (A.2.1), and at various temperatures with different inoculum doses (A.2.2). B = Complex biotopes including the presence of flora/fauna, at 25°C (B.1) and other temperatures (B.2).

^b^ Avian strain stands for the A/Chicken/Cambodia/LC1AL/2007 strain. Human strain stands for the A/Cambodia/408008/2005 strain.

^c^ T° = Temperature (°C).

*last day of the corresponding experiment at which samples could be collected and tested.
