# Supplementary material for: Highly Pathogenic Influenza A(H5N1) Virus Survival in Complex Artificial Aquatic Biotopes
Source: PLoS One. 2012 Apr 13;7(4):e34160. doi: 10.1371/journal.pone.0034160 (PMC3325971; doi:10.1371/journal.pone.0034160)
Supplement: Table S5 — Viral load (number of H5 RNA copies/g) measured in different mussel organs obtained in experiment C.2 (virus A/Cambodia/408008/2005). (DOC) [file pone.0034160.s006.doc]

**Supplementary Table 5. Viral load (number of H5 RNA copies/g) measured in different mussel organs obtained in experiments C.2 (virus A/Cambodia/408008/2005).**

|  |  | **Mussel organs** | | | |
| --- | --- | --- | --- | --- | --- |
|  | **Day of incubation** | **Gills** | **Intestine** | **Digestive gland** | **Remaining carcass** |
| **Mussel immersed in contaminated water (M-Inf^a^) (experiment C.2.a)** | M-Inf^a^* Day 1 | 1.70×10^6^ | 2.30×10^5^ | 3.20×10^4^ | 2.40×10^5^ |
|  | M-Inf^a^* Day 2 | 2.20×10^5^ | 9.94×10^4^ | 1.73×10^4^ | 4.91×10^3^ |
|  | M-Inf^a^* Day 3 | 2.70×10^5^ | 6.96×10^2^ | 3.27×10^5^ | 1.27×10^5^ |
|  | M-Inf^a^* Day 4 | 7.50×10^2^ | 2.10×10^4^ | 1.14×10^4^ | 7.38×10^4^ |
|  | M-Inf^a*^ Day 5 | 3.23×10^3^ | 1.09×10^3^ | 6.97×10^2^ | 3.74×10^2^ |
|  | M-Inf^a*^ Day 6 | Negative | Negative | Negative | 1.46 × 10^4^ |
|  | M-Inf^a^ Day 7 | 7.21×10^3^ | 1.85×10^3^ | 9.37×10^2^ | 5.79×10^3^ |
|  | M-Inf^a^ Day 8 | 2.30×10^4^ | 2.10×10^3^ | 1.14×10^4^ | 1.15×10^4^ |
| **Mussel kept 4 days in contaminated water then transferred to non-contaminated water (M-Inf^b^) (experiment C.2.b)** | M-Inf^b^ Day 5 | 7.37×10^2^ | 1.04×10^3^ | 1.36×10^3^ | Negative |
|  | M-Inf^b^ Day 6 | Negative | Negative | Negative | Negative |
| **Mussel kept 4 days in contaminated water, 1 day in non-contaminated water then transferred to non-contaminated water (M-Inf^c^) (experiment C.2.c)** | M-Inf^c^ Day 6 | 4.20×10^3^ | 2.80×10^5^ | Negative | 5.69×10^3^ |
|  | M-Inf^c^ Day 7 | 1.39×10^4^ | 8.28×10^3^ | 5.95×10^3^ | 9.70×10^4^ |
|  | M-Inf^c^ Day 8 | 6.43×10^2^ | Negative | 5.35×10^2^ | 2.85×10^3^ |
| **Mussel exposed (M-Exp) to contaminated mussel (M-Inf^c^) (experiment C.2.c)** | M-Exp Day 6 | 1.13×10^3^ | 5.80×10^1^ | 3.47×10^3^ | 4.58×10^3^ |
|  | M-Exp Day 7 | 2.04×10^4^ | 2.74×10^4^ | 1.14×10^4^ | 3.43×10^5^ |
|  | M-Exp Day 8 | Negative | Negative | Negative | Negative |

* Mussel in which infectious virus particles were detected
