# Supplementary material for: Highly Pathogenic Influenza A(H5N1) Virus Survival in Complex Artificial Aquatic Biotopes
Source: PLoS One. 2012 Apr 13;7(4):e34160. doi: 10.1371/journal.pone.0034160 (PMC3325971; doi:10.1371/journal.pone.0034160)
Supplement: Table S6 — Viral load (number of H5 RNA copies/g) in different fish and tadpole organs obtained from experiments D (virus A/chicken/Cambodia/LC1AL/2007). (DOC) [file pone.0034160.s007.doc]

**Supplementary Table 6. Viral load (number of H5 RNA copies/g) in different fish and tadpole organs obtained from experiments D (virus A/chicken/Cambodia/LC1AL/2007)**

|  |  | **Organs** | | | | | |
| --- | --- | --- | --- | --- | --- | --- | --- |
|  | **Day of incubation** | **Gills** | **Intestine** | **scales** | **Fins** | **Brain** | **Remaining carcass** |
| **Fish immersed in contaminated water (F-Inf^a^)**  **(experiment D1)** | F-Inf^a^* Day1 | 4.73×10^6^ | 1.85×10^4^ | 4.04×10^4^ | 1.09×10^5^ | 1.85×10^5^ | 3.37×10^3^ |
|  | F-Inf^a^ Day3 | 1.05×10^6^ | 3.84×10^4^ | 4.34×10^3^ | 1.69×10^6^ | 4.76×10^3^ | 4.24×10^3^ |
|  | F-Inf^a^ Day5 | 2.72×10^4^ | 1.00×10^5^ | 3.94×10^4^ | 1.62×10^6^ | 1.10×10^6^ | 2.10×10^5^ |
|  | F-Inf^a^ Day7 | 2.82×10^4^ | 2.93×10^6^ | 1.03×10^4^ | 1.17×10^4^ | 1.08×10^5^ | 5.85×10^5^ |
|  | F-Inf^a^ Day11 | 1.83×10^6^ | 7.02×10^5^ | 3.69×10^4^ | 1.21×10^5^ | 4.11×10^5^ | 5.09×10^4^ |
|  | F-Inf^a^ Day15 | 7.49×10^5^ | 1.19×10^5^ | 7.55×10^4^ | 1.00×10^5^ | 6.74×10^4^ | 1.54×10^4^ |
|  | F-Inf^a^ Day20 | Negative | 5.42×10^3^ | 1.48×10^4^ | Negative | Negative | Negative |
| **Tadpole immersed in contaminated water (T-Inf)**  **(experiment D1)** | T-Inf* Day1 | 2.80×10^7^ | 6.83×10^6^ | NA | NA | NA | 1.79×10^7^ |
|  | T-Inf Day13 | 1.53×10^5^ | 2.72×10^5^ | NA | NA | NA | 5.82×10^5^ |
|  | T-Inf Day14 | 1.12×10^5^ | 9.83×10^4^ | NA | NA | NA | 1.55×10^5^ |
| **Fish from contaminated water transferred to non-contaminated water (F-Inf^b^)**  **(experiment D2)** | F-Inf^b^ Day7 | 3.60×10^6^ | 5.50×10^6^ | 2.80×10^5^ | 1.90×10^3^ | 1.37×10^7^ | 2.01×10^3^ |
| **Fish exposed to contaminated fish (F-exp)**  **(experiment D2)** | F-Exp Day7 | 6.60×10^3^ | 3.40×10^4^ | 1.64×10^4^ | 2.66×10^5^ | 1.60×10^6^ | 1.40×10^5^ |

* Detection of infectious virus particles

NA: not available (no organ tested).
