# Supplementary material for: Highly Pathogenic Influenza A(H5N1) Virus Survival in Complex Artificial Aquatic Biotopes
Source: PLoS One. 2012 Apr 13;7(4):e34160. doi: 10.1371/journal.pone.0034160 (PMC3325971; doi:10.1371/journal.pone.0034160)
Supplement: Table S7 — Survival of infectious particles and persistence of virus RNA in aquatic environments: compiled data. (RTF) [file pone.0034160.s008.rtf]

Supplementary Table 7: Survival of H5N1 infectious particles and persistence of viral RNA in aquatic environments: compiled data.

Series #a	Virus originb	Viral concentration (EID50/mL water)	T°	Water origin	Survival of infectious particles in water (days)	Persistence of viral RNA in water (days)	N# of viral RNA copies /mL of water	Mud origin	Survival of infectious particles in mud  (days)	Persistence of viral RNA in mud (days)	N# of  RNA copies /g of mud	Flora/fauna	Survival of infectious particles in flora/fauna (days)	Persistence of viral RNA in flora/fauna (days)	N# of RNA copies /g	
A.1	Avian	5104	25	Pond 1	0	5	2.00100	NA	NA	NA	NA	NA	NA	NA	NA	
				Pond 2	0	5	8.00100	NA	NA	NA	NA	NA	NA	NA	NA	
				Rain	4	9	1.30101	NA	NA	NA	NA	NA	NA	NA	NA	
	Human	5104	25	Pond 1	0	4	2.22100	NA	NA	NA	NA	NA	NA	NA	NA	
				Pond 2	0	3	3.96101	NA	NA	NA	NA	NA	NA	NA	NA	
				Lake	0	11	2.22102	NA	NA	NA	NA	NA	NA	NA	NA	
				Rain	0	4	4.20100	NA	NA	NA	NA	NA	NA	NA	NA	
A.2.1	Avian	5104	25	Pond 1	0	5	3.50100	Pond 1	0	12	1.26102	NA	NA	NA	NA	
				Pond 2	0	5	9.16101	Pond 2	0	14*	2.77103	NA	NA	NA	NA	
				Lake	0	1	1.55102	Lake	0	13	1.26104	NA	NA	NA	NA	
A.2.2	Avian	5102	22	Pond 2	0	5	1.82102	Pond 2	0	14*	1.79103	NA	NA	NA	NA	
			25	Pond 1	0	14*	5.20101	Pond 1	0	14*	2.23102	NA	NA	NA	NA	
			32	Pond 2	0	4	1.78101	Pond 2	0	14*	5.50101	NA	NA	NA	NA	
			34	Pond 1	0	6	2.02102	Pond 1	0	14*	1.53102	NA	NA	NA	NA	
	Human	5103	25	Lake	0	7	3.22100	Lake	0	6	1.20103	NA	NA	NA	NA	
			32	Lake	0	6	1.05101	Lake	0	6	4.30103	NA	NA	NA	NA	
B.1	Avian	5102	25	Pond 1	0	4	1.28101	Pond 1	0	14*	2.60102	Plants	0	0	0	
												Guppies	0	0	0	
												Snails	0	0	0	
												Clams	0	0	0	
		5104	25	Lake	0	5	1.56101	Lake	0	14*	1.30104	Plants	0	1	1.20104	
												Guppies	0	0	0	
												Snails	0	0	0	
												Clams	0	3*	5.70104	
												Mussels	0	2	3.30103	
	Human	5103	25	Lake	0	7	9.02100	Lake	0	8	1.09103	Plants	0	0	0	
												Guppies	0	3	9.33103	
												Tadpoles	0	3	8.08103	
		5104	25	Lake	0	2	4.75101	Lake	0	1	1.60104	Plants	0	0	0	
												Guppies	0	0	0	
												Clams	0	0	0	
B.2	Avian	5102	22	Pond 2	0	2	1.70103	Pond 2	0	14*	3.05102	Plants	0	0	0	
												Guppies	0	0	0	
												Snails	0	0	0	
												Clams	0	9*	9.08102	
			32	Pond 2	0	7	7.88102	Pond 2	0	10	1.29102	Plants	0	0	0	
												Guppies	0	0	0	
												Snails	0	0	0	
												Clams	0	0	0	
			34	Pond 1	0	3	1.11101	Pond 1	0	14*	4.45102	Plants	0	0	0	
												Guppies	0	0	0	
												Snails	0	0	0	
												Clams	0	0	0	
	Human	5103	32	Lake	0	3	1.09101	Lake	0	6	1.13103	Plants	0	0	0	
												Guppies	0	3	5.29104	
												Tadpoles	0	3	1.59104	
C	Human	5104	25	Rain	4	9*	5.50102	NA	NA	NA	NA	Mussels	6	8*	2.25104	
D	Avian	2102	18-22	Rain	2	20*	2.10104	NA	NA	NA	NA	Tadpoles	1	14*	1.12106	
												Fighting fish	1	20*	1.48104	
a Series numbers as defined in Table 1. A = Simple biotopes, with A.1 = only water, no mud, A.2 = water and mud at 25°C with the standard inoculum dose of 5104 EID50/mL water (A.2.1), and at various temperatures with different inoculum doses (A.2.2). B = Complex biotopes including the presence of flora/fauna, at 25°C (B.1) and other temperatures (B.2).
b Avian strain stands for the A/Chicken/Cambodia/LC1AL/2007 strain. Human strain stands for the A/Cambodia/408008/2005 strain.
T° = Temperature (°C).
*last day of the corresponding experiment at which samples could be collected and tested.
